# Shared Decision-Making for Nursing Practice: An Integrative Review

**DOI:** 10.2174/1874434601812010001

**Published:** 2018-01-22

**Authors:** Marie Truglio-Londrigan, Jason T. Slyer

**Affiliations:** 1 Pace University, College of Health Professions, Lienhard School of Nursing 861 Bedford Road Pleasantville, NY 10570, USA; 2 Clinical Assistant Professor, Pace University, College of Health Professions, Lienhard School of Nursing 163 William Street, 5^th^ Floor New York, NY 10036, USA

**Keywords:** Shared decision-making, Nurse-patient relationship, Reflection, Communication, Integrative review, Practice model

## Abstract

**Background:**

Shared decision-making has received national and international interest by providers, educators, researchers, and policy makers. The literature on shared decision-making is extensive, dealing with the individual components of shared decision-making rather than a comprehensive process. This view of shared decision-making leaves healthcare providers to wonder how to integrate shared decision-making into practice.

**Objective:**

To understand shared decision-making as a comprehensive process from the perspective of the patient and provider in all healthcare settings.

**Methods:**

An integrative review was conducted applying a systematic approach involving a literature search, data evaluation, and data analysis. The search included articles from PubMed, CINAHL, the Cochrane Central Register of Controlled Trials, and PsycINFO from 1970 through 2016. Articles included quantitative experimental and non-experimental designs, qualitative, and theoretical articles about shared decision-making between all healthcare providers and patients in all healthcare settings.

**Results:**

Fifty-two papers were included in this integrative review. Three categories emerged from the synthesis: (a) communication/ relationship building; (b) working towards a shared decision; and (c) action for shared decision-making. Each major theme contained sub-themes represented in the proposed visual representation for shared decision-making.

**Conclusion:**

A comprehensive understanding of shared decision-making between the nurse and the patient was identified. A visual representation offers a guide that depicts shared decision-making as a process taking place during a healthcare encounter with implications for the continuation of shared decisions over time offering patients an opportunity to return to the nurse for reconsiderations of past shared decisions.

## 1 INTRODUCTION

Shared decision-making (SDM) has received national and international attention from providers, educators, researchers, and policy makers [1-5]. Shared decision-making has been described as taking place in a relationship where there is a partnership between the provider and the patient characterized by a collaborative bi-directional mutual exchange of information and discussion involving negotiation leading to a shared decision [6]. Shared decision-making, therefore, takes place in a relationship that is participatory, collaborative, open, and respectful. The relationship is one in which there are at least two participants: the nurse, as the provider, and the patient. Trust and respect between providers and patients has also been described as foundational for SDM [7-10].

The literature on SDM is extensive. These works describe the individual components of SDM, including the facilitators and barriers to the achievement of SDM [10-14]. Provider SDM competencies have also been explored in the literature [15-17] along with the context of the provider and patient relationship such as the need for resources, including time [6, 18-29]. Research has also been conducted to examine the effect of SDM on patient outcomes with regard to chronic and acute illnesses [20, 27, 30, 31]; treatment adherence [31]; patient coping [32, 33]; knowledge attainment and empowerment [34, 35]; autonomy and self-determination [22, 36, 37]; and, patient satisfaction [26, 38, 39]. Despite this research, the overall evidence as to the effect of SDM leading to positive patient outcomes is inconclusive [40, 41].

Nurses develop relationships and work with individuals, families, communities and populations across diverse healthcare settings. Hildegard E. Peplau [42] provided a framework for the nursing professions’ understanding of the nurse-patient helping relationship as the nexus from which there is growth. Millard, Hallett and Luker [43] saw the importance of the nurse-patient relationship as the vehicle for the exchange of information necessary for SDM and suggested that there is a need for nurses to “pay attention to the quality and nature of the relationships they have with their patient” [43]. Furthermore, Clark, *et al.* [40] examined the nurse-patient dyad as an intervention necessary to both sustain the relationship and facilitate SDM towards care management.

The focus on SDM has been on the dyad relationship and the individual components of SDM rather than describing and explaining the process taking place within the relationship. Gulbrandsen [44] noted that the contemporary literature’s portrayal of SDM does not do an adequate job of illustrating the processes of SDM. A comprehensive understanding of SDM as a process would be meaningful for nurses as they work with patients towards shared decisions about care management.

## 2 AIM

The aim of this integrative review is to understand the comprehensive process of SDM from the perspective of the patient and provider in all healthcare settings. Understanding the process will create a common language and appreciation of SDM for meaningful nursing practice [45].

## 3 METHODS

This integrative review applied the comprehensive and systematic approach described by Whittemore and Knafl [46] consisting of the literature search, data evaluation, and data analysis. This method facilitated the gathering of information and research from a variety of methodologies (quantitative, qualitative, and theoretical) supporting an integrative approach allowing for a comprehensive depiction of the process of SDM.

### 3.1 Inclusion Criteria

Articles considered for inclusion were qualitative or quantitative research articles or theoretical literature that addressed SDM taking place within a relationship between the patient and the provider. Patients needed to be 18 years of age or older and providers could represent any healthcare field. Only articles published in English were considered. Articles were excluded if they solely addressed intervention strategies such as education to enhance SDM competencies in providers or decision aids as an intervention to assist patients in their shared decision rather than a focus on the process of SDM taking place in a relationship. Articles focusing on shared decision-making in psychiatric or mental health settings were excluded because of the unique issues within this patient population pertaining to SDM.

### 3.2 Search Strategy

A comprehensive literature search was applied in PubMed, CINAHL, the Cochrane Central Register of Controlled Trials (CENTRAL), and PsycINFO. Diverse literature available in English was searched from 1970 through January 2016, including quantitative designs (both experimental and non-experimental), qualitative designs, and theoretical papers. Three searches were conducted in each database in order to identify literature related to SDM inclusive of the patient, the provider, and the environment. (Table **1**) depicts these basic search strategies along with the key terms used.

### 3.3 Data Evaluation

Articles that met the inclusion criteria were evaluated for methodological quality. The standardized critical appraisal instruments for experimental, observations, quantitative descriptive, qualitative, and expert opinion/theoretical works from the Joanna Briggs Institute System for the Unified Management, Assessment and Review of Information (JBI-SUMARI) were used for this assessment [47]. This stage reduced the possibility of bias and errors by including only papers deemed reliable/dependable and valid/credible [47]. Any disagreements between the reviewers were resolved through discussion until consensus was reached. Supplemental (Table **S1**) contains the results of the critical appraisals for all included studies.

### 3.4 Data Analysis

Data analyses were carried out through the application of an inductive content analysis process that involved creating categories and abstractions [48]. The categories were then further grouped under higher order headings [48, 49]. The synthesis process involved creating categories that describes all of the aspects of the SDM process leading to a new representation of facts offering a visual representation of SDM as a guide for nursing practice [50].

## 4 RESULTS

**Fig. (1):**
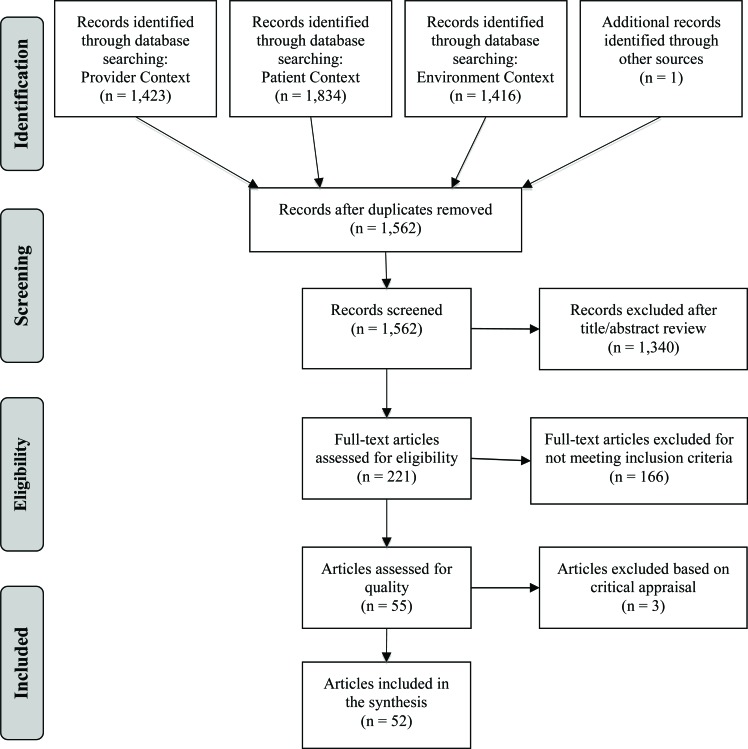
PRISMA Flow Diagram [54].

Upon completion of the initial searches, 4,674 potentially relevant titles were identified. Duplicates were removed, leaving 1,562 articles for review. After reviewing the titles and abstracts, 1,340 articles were excluded for not meeting the inclusion criteria. After full text review, an additional 166 articles were excluded for not meet the inclusion criteria, leaving 55 articles for critical appraisal. Three articles were excluded for methodological weaknesses in the research and limited results sections (Fig. **1**) [51-53].

Fifty-two articles published between 1997 and 2016 were included in this review. Supplemental Table (**S2**) contains an overview of the included articles. Twenty-three of the articles originated from the United States, six from the United Kingdom, five from Germany, nine from Canada, two from the Netherlands, and one each from Australia, Denmark, Norway, Italy, and France. Two articles originated from multiple countries. Sixteen of the articles were quantitative designs, 19 were qualitative, one was mixed method, and 16 were conceptual.

The analysis of this integrative review and the articles retained from data analysis generated three categories: (a) communication/relationship building; (b) working towards a shared decision; and (c) action for SDM, each containing sub-themes that depict the process of SDM. (Table **2**) outlines the three categories and sub-categories along with the corresponding articles informing each category. These categories and sub-categories were further contextualized into a visual representation of the shared decision-making process seen in Fig. (**2**).

### 4.1 Communication and Relationship

Communication and relationship building is the first general category and is foundational for the SDM process. The three sub-themes within this theme are: relationship building—trust and respect; information exchange— communication; and context.

#### 4.1.1 Relationship Building—Trust and Respect

Individuals enter into the relationship and must work towards building a trusting and respectful relationship where SDM is invited and encouraged. The work begins as the patient identifies a need or question. This need and/or question influences the patient’s quest for answers [76]. The relationship is the vehicle by which providers and patients “act in a relational way” and the individuals are “actively seeking a personal connection with each other” [65]. The relationship is a partnership where there is collaboration and a sharing of power [30, 34]. With the sharing of power, there is mutual responsibility toward one another [16]. The relationship is strengthened over time leading to bi-directional trust and respect [58, 67]. Patients who feel trusted and respected are more open and share information with their provider thereby facilitating communication for SDM [13].

#### 4.1.2 Information Exchange—Communication

Information exchange *via* interpersonal and intrapersonal communication sustains the relationship. The *interpersonal process* of communication is bi-directional between the provider and the patient when there is a mutual exchange of information [6, 19, 22, 23, 25, 38, 67, 70, 72]. The exchange of information also involves active listening [29, 63, 69]. Emotions such as fear, anger, and anxiety can interfere with a patient’s readiness to communicate [12, 14]. Furthermore, a provider’s readiness and receptiveness to explore a patient’s feelings and preferences is important [65]. For example, the emotional tone the provider creates facilitates an atmosphere of compassion and caring that enhances open communication [11, 71]. In situations where this emotional tone is not created the patient is less likely to feel compassion or care and may perceive the provider’s approach as “authoritarian.” This perception may prompt the patient’s reluctance to communicate and establishing a “shield” –creating a barrier to SDM [21].

The *intrapersonal process* of communication also plays a role in the achievement of SDM taking place *within* the provider and patient through the process of reflection [73]. The reflection process takes place at two levels. Mutual reflection takes place when the provider and the patient reflect together *via* communication, exchanging thoughts about decisions, and clarifying the patient’s perspective [73]. Individual reflection takes place autonomously within the individual provider or patient [73]. For example, during an individual reflective moment a provider may identify “blind spots” in a patient’s perception of an experience which may be limiting the patient’s insight about an issue [73]. During the corresponding mutual reflection, the provider uses communication skills to challenge the patient verbally and non-verbally while encouraging the patient to also engage intrapersonal self-reflection. The mutual reflection process, therefore, encourages patients to engage in their own independent reflections that helps them recognize “a new decision or a new position on the difficulty or challenge on which they had been reflecting” [73]. Furthermore, providers and patients continually reflect upon their relationship and communication over time known as post-decision deliberation. These deliberations offer an opportunity for reconsideration of past decisions illustrating the on-going process of decision-making [68].

#### 4.1.3 Context

The provider and the patient work within a particular healthcare context that either facilitates or creates barriers for SDM. From the patient’s perspective, the context includes the patient’s family, friends, and home, including community supports and networks [24, 29]. For example, patients who are accompanied by family members to healthcare encounters are more likely to engage in SDM [21, 26, 27]. The context of the provider’s work environment also influences their ability to integrate SDM into practice [20, 22, 23, 28]. Time and access to resources are facilitators for SDM [6, 18, 25, 52]. Organizational models and systems that facilitate patients’ access to their provider(s) and/or healthcare team reduce fragmentation and improve collaboration, coordination, and SDM [25]. Technology capable of tracing patients’ progress through the SDM process is a valuable resource [19, 21]. Shared decision-making is prominent in the thoughts of healthcare providers within the larger healthcare system; however, so too are evidence-based practice (EBP) and clinical practice guidelines. The challenge for providers is to ensure that the realities of clinical practice are addressed along with the patient’s preferences [25].

### 4.2 Work Toward Shared Decision-Making

Communication and relationship building are foundational for the initiation of SDM. Shared decision-making, however, requires dedicated ongoing work. The second general category, work towards SDM, has four sub-categories: assessment, teaching-learning, finding balance, and decision.

#### 4.2.1 Assessment

The work towards SDM begins with an assessment. The assessment of the individual is foundational as the provider must “come to know one’s patient” [29] and the patient’s specific preferences [35]. Understanding the individual patient characteristics begins with an awareness of the patient’s age, gender, race, spiritual and cultural beliefs, education, and life experiences. All of these characteristics influence the patient’s beliefs about SDM and the value placed on SDM [35, 63]. For example, the assessment will reveal whether patients see themselves as sharing in decision-making, or whether they prefer the provider to be the primary decision maker? The role a patient chooses to play may change over time, depending on the situation for which the patient is seeking assistance [74]. Furthermore, as the work towards the shared decision takes place, there will be moments when the provider’s expertise will warrant that they take the lead in the encounter and other moments when the patient will take the lead [74]. Race too may influence a patient’s behavior if an individual decides not to share information for reasons of racially inspired mistrust [58, 59]. Age may influence behaviors as research has shown that younger individuals choose to engage in SDM compared to older adults [26, 28]. This is also true of individuals with higher levels of education and literacy [14, 25, 60].

The assessment continues as the provider asks questions about the reasons the patient is seeking assistance. How SDM unfolds varies depending upon the acuity or chronicity of illness [27, 28, 73, 75]. Acute illness may foster a provider-led approach to SDM. Conversely, chronic illness fosters a patient-led approach with patients who are responsible for the self-management of their illness over time in their own home/community, often with the support of family or friends [27, 29, 39]. Gathering information about social support and social networks, therefore, is a part of the assessment [29] as these networks have been found to facilitate a patient’s ability to be active and engaged in SDM [6, 26]. Ultimately, the assessment offers the provider an opportunity to know the patient, the patient’s family, and home/community, building a practice based on facts and evidence not assumptions.

#### 4.2.2 Teaching-Learning

Shared decision-making warrants that patients have the necessary information that they need to know so that they can share in the decision-making process [78]. Providers, therefore, will need to teach and provide their patients with this information. What providers teach to support learning depends on the assessment [27, 28, 33, 74]. For example, the provider needs to consider the readiness of the patient and the amount and type of information that needs to be taught and how to best teach that information for a specific patient [21]. This is vital in today’s EBP-driven healthcare environment. The EBP process involves sharing information with the patient about diagnosis and treatment, educating the patient about the disease and treatment options, and informing the patient about the strength of the evidence, as well as the risks, benefits, and possible outcomes [68]. Information gathered during the assessment guides providers so that they are mindful of a patient’s age, literacy, language, and culture in the development and delivery of educational information. Patient-centered education applies specific teaching strategies for specific patients, such as culturally appropriate decision aids, which both guide patient learning and facilitate the patient’s understanding of information [15, 27, 29, 60, 68, 77].

#### 4.2.3 Finding Balance

Providers and patients come together due to identified needs/issues. A need/issue causes uncertainty [76] and challenges providers and patients to find a resolution through SDM. Part of the work of SDM is achieving balance necessary to arrive at a shared decision [20, 37]. This is especially relevant with regard to EBP. For example, in a clinical practice scenario when there is evidence that there may be alternative best practice choices, the provider’s competence in the use of equipoise in the search for a balanced shared decision is sought. The concept of equipoise is exemplified by “talk” where there is the presentation of information, portrayal of options and exploitation of alternatives, as well as deliberation [15].

What happens, however, in situations where there is no documented evidence for best practice or there is only one best practice choice that a patient considers unacceptable because of personal ideas, values, or beliefs? These encounters invoke the ethics of practice, including the principles of autonomy and beneficence. The provider and the patient together seek to achieve balance between these principles through the application of skills such as talking, openness, and information provision [28, 31, 36, 77]. Furthermore, part of the work in finding balance requires deliberation and negotiation leading to consensus about the decision [6, 37, 65, 69].

#### 4.2.4 The Decision

Communication and relationship building, assessment, teaching and learning and the seeking of balance are all part of the SDM process leading to consensus about the decision. The work is individual for every patient and facilitates care that is patient-centered [6, 20, 35, 65, 69, 74]. Ultimately, the shared decision is not the end point but signals the need for the patient to take action and carry out the decision.

### 4.3 Action for Shared Decision-Making

This third theme, action for SDM, contains two sub-themes: Takes action or no action.

#### 4.3.1 Takes Action

Shared decision-making does not end with the decision. Once the provider and patient come to a shared decision there needs to be action by the patient. The process of SDM, therefore, moves beyond the decision point as the patient engages in the steps necessary to take action to see the decision through [73]. For example, patients return to their homes/communities where they attempt to carry out their decisions. During this process, the implementation of the decision may be seamless, the patient is satisfied, and the issue or question is addressed. There may be, however, times when patients find the action challenging or the actions that are required are not what was expected. In these situations, the patient may not be satisfied resulting in an unresolved issue or questions prompting the patient to return to the provider to re-evaluate the decision [13, 19, 21, 27, 63, 68, 73].

#### 4.3.2 No Action

No action occurs when patients return to their homes/communities; however, once in their familiar environment, they chose not to initiate the steps and actions to see their decisions through. For example, patients may feel pressured by the perceived power imbalance they experienced with their provider and as a result found themselves aligning with a particular decision favored by the provider [21, 34]. As a result, when patients return to their homes/communities they choose not to act. This realization may trigger the need to return to the provider or in some cases a patient may choose not to return for further care [13, 19, 21, 27, 63, 68, 73].

## 5 DISCUSSION

The significance of this integrative review is noted in the presentation of the ongoing process of SDM. Box (**1**) below provides a case study that exemplifies this ongoing process. This process takes place in practice between a nurse and patient during a healthcare encounter where there is an identified need/issue or question. The *relationship* is one of a partnership where both parties are collaborating. The relationship that develops is one where trust and respect is fostered by the communication between the nurse and the patient. *Communication* is both interpersonal and intrapersonal. Interpersonal communication between the nurse and patient takes place during the healthcare encounter. Intrapersonal communication takes place during the encounter when the nurse and patient think about—*via* *reflection*—what they are saying, doing, and observing at the moment they are actively engaged [79]. For example, a nurse may reflect on a patient’s non-responsiveness to a conversation. Nurses who are knowledgeable about communication and skillful in the application of communication techniques will use strategic questioning where options are explored and listening to facilitate a patient’s insight into the presiding issue [80]. Reflection also continues after the interaction as nurses and patients reflect upon past SDM healthcare encounters. During these moments, patients may have questions and/or decide that the initial decision is no longer acceptable and wish to return to their nurse. This review highlights relationship building and communication in nursing practice that is foundational for SDM and signals that communication is complex, requiring nurses to be ever vigilant about what they are saying and doing, as well as the patient’s response. Being aware of one’s own reflections as well as one’s skills to assist patients in their own self-reflection facilitates a practice based in SDM. In addition, this review highlights the need for a practice environment that fosters relationships and communication by establishing practice models where ongoing connections between the nurse and patient are consistent and continuous, thereby supporting and sustaining SDM.

*Flexibility* in the nurse-patient relationship is identified as significant in this review and takes place as nurses and patients work together, alternating who takes the lead during SDM. There may be times when the nurse takes the lead to educate the patient about best practices while considering patient characteristics and the patient’s response to the information. As the work continues, the patient may take the lead, being the expert in his/her own life experiences. Flexibility in the SDM process also takes place in the bi-directional communication between the nurse and the patient as discussions take place about EBP. These discussions are a give and take of ideas about EBP and choices about treatments; when balance is achieved, a shared decision can be reached.

This review also highlights the need for nurses to be continually aware of the importance of *context* in the form of family/friends, community, organization, and the greater healthcare system. For example, practice models that are intra and interprofessionally based will enhance patients’ access to available organizational providers in the event they need to return to re-evaluate a past decision. These practice models also enrich the support, guidance, teaching, and mentoring of patients [23, 25, 27, 29]. Resources that foster and facilitate SDM such as time, consultation services, reliable and valid decision aids that are culturally appropriate, and clinical information systems that track a patient’s progress in the achievement of shared decisions are necessary. These examples suggest policy changes at the organizational level. At the healthcare system level, the development of standards of practice based in evidence, while beneficial, have been viewed as a challenge by others as there may be the potential for “fewer choices being offered to patients by healthcare providers” [25].

*Education* initiatives that enhance the nurse’s ability to integrate SDM into their practice are significant. Competencies need to be achieved in the area of reflective practice, the nurse-patient relationship, communication and strategic questioning, assessment, teaching and learning, ethics, and the role of social supports and social networks within a community. Part of this educational endeavor also includes nurses examining their own comfort levels about SDM. For example, nurses may express positive beliefs about SDM; however, these beliefs may not manifest in practice as the nurse may be ambivalent about a partnership with a patient due to a lack of trust in a patient’s ability [16, 64]. Patents too will need to be competent in order to be active and engaged in the SDM process. Their competency, however, is centered around the information that they need to know to participate in SDM. This means that the SDM encounter will require that nurses provide support, guidance, mentoring, coordination, and education to patients throughout the entire SDM process. Nurses, therefore, will need to assume a diverse set of roles beyond caregiver as they adjust to the flexible nature of SDM. For example, the shared decision may require a course of action in which the patient needs to access community resources. Nurses will educate patients on what community resources are available, offer advice and support patients as they access services, and advocate when a patient has difficulty connecting with these services.

The visual representation of SDM Fig. (**2**) offered in this review provides nurses with a guide for practice and also for research. Contemplating the guide offers cues for hypothesis generation and the raising of qualitative questions that will add to the body of nursing knowledge. For example, there is limited information in the literature about patients returning to their home/communities as they attempt to take the necessary steps and carry out the actions for the shared decision. The development of qualitative descriptive studies to describe what happens as patients attempts to initiate shared decisions once they leave a healthcare encounter would provide valuable evidence for nurses as they address needed practice changes to facilitate SDM.

## CONCLUSION

Shared decision-making has received attention in the recent years, however, this attention has focused on the individual components of SDM rather than a comprehensive process. An understanding of SDM that captures this comprehensive process would facilitate SDM in practice, research, and the development of educational programs for nurses and other healthcare providers that embrace all aspects of the process. To this end, an integrative review was conducted applying the systematic approach described by Whittemore and Knafl [46]. The outcome of this integrative review provides an understanding of SDM as a comprehensive process that takes place between the nurse and the patient. It provides an opportunity to consider the complexity of SDM as an on-going process that does not end with the decision. The visual representation is a guide that depicts the processes of SDM taking place during the healthcare encounter with implications for the shared decision over time in the event a patient needs to return to the nurse to reconsider earlier decisions.

## Figures and Tables

**Fig. (2):**
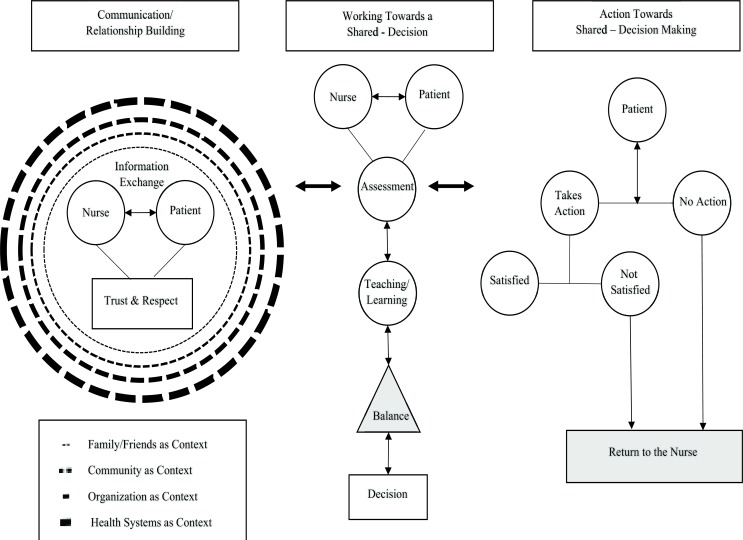
A visual representation for shared decision-making in practice.

**Table 1:** Basic search term strategies used across all databases.

**Patient search:**				
Shared decision making	AND	Patient or patients or client or clients	AND	Experience or experiences or perspective or perspectives or satisfaction or preference or preferences or competent or competency or competencies or demographics or diagnosis or outcome or outcomes or literacy or culture or education
**Provider search:**				
Shared decision making	AND	Physician or physicians or doctor or doctors or clinician or clinician or provider or providers or nurse or nurses	AND	Experience or experiences or perspective or perspectives or satisfaction or preference or preferences or competent or competency or competencies or demographics or diagnosis or outcome or outcomes or literacy or culture or education
**Environment search:**				
Shared decision making	AND	Environment or “environmental culture” or organization or “organizational culture” or policy or “health service culture” or context or commitment or consistency or continuity or time or economics or “financial resources” or resources		

**Table 2:** Shared decision-marking categories and subcategories.

Category	Sub-category	References
Communication and relationship building	Individual characteristics	Peek, *et al.* [14], Towle, *et al.* [16], Friedberg, *et al.* [19], Légaré, *et al.* [24], Légaré and Witteman [25], Mandelblatt, *et al.* [26], Muthalagappan, *et al.* [28], Bieber, *et al.* [32], Sacchi, *et al.* [35], Bernhard, Butow, Aldridge, *et al.* [55], Charles, Gafni, Whelan, *et al.* [56], Isaacs, Kistler, Hunold, *et al.* [57], Peek, Gorawara-Bhat, Quinn, *et al.* [58], Peek, Odoms-Young, Quinn, *et al.* [59], Shalowitz and Wolf [60], Smith, Juraskova, Butow, *et al.* [61], Tinsel, Buchholz, Vach, *et al.* [62], Truglio-Londrigan [63], Upton, Fletcher, Madoc-Sutton, *et al.* [64]
	Relationship building—trust and respect	Durif-Bruckert, *et al.* [13], Towle, *et al.* [16], Muthalagappan, *et al.* [28], Deinzer, *et al.* [30], Hain and Sandy [34], Peek, *et al.* [58], Lown, Clark and Hanson [65], Ommen, Thuem, Pfaff, *et al.* [66], Shay and Lafata [67]
	Information exchange—communication	Charles, *et al.* [6], Bot, *et al.* [11], Charles, *et al.* [12], Peek, *et al.* [14], Edwards, *et al.* [18], Friedberg, *et al.* [19], Frosch, *et al.* [21], Hess, *et al.* [22], Lally, *et al.* [23], Légaré and Witteman [25], Mandelblatt, *et al.* [26], [27], Truglio-Londrigan [29], Bieber, *et al.* [32], Glass, *et al.* [38], Truglio-Londrigan [63], Lown, *et al.* [65], Shay and Lafata [67], Ford, Schofield and Hope [68], Saba, Wong, Schillinger, *et al.* [69], Siminoff and Step [70], Thorne, Oliffe and Stajduhar [71], White, Keller and Horrigan [72], Zoffmann, Harder and Kirkevold [73]
	Context	Charles, *et al.* [6], Edwards, *et al.* [18], Friedberg, *et al.* [19], Friesen-Storms, *et al.* [20], Frosch, *et al.* [21], Hess, *et al.* [22], Lally, *et al.* [23], Légaré, *et al.* [24], Légaré and Witteman [25], Mandelblatt, *et al.* [26], Montori, *et al.* [27], Muthalagappan, *et al.* [28], Truglio-Londrigan [29]
Work toward shared decision-making	Assessment	Charles, *et al.* [6], Légaré, *et al.* [24], Mandelblatt, *et al.* [26], Montori, *et al.* [27], Muthalagappan, *et al.* [28], Truglio-Londrigan [29], Wilson, *et al.* [31], Shabason, *et al.* [39], Zoffmann, *et al.* [73], Charles, Gafni and Whelan [74], Müller-Engelmann, Keller, Donner-Banzhoff, *et al.* [75]
	Finding balance	Charles, *et al.* [6], Elwyn, *et al.* [15], Friesen-Storms, *et al.* [20], Hess, *et al.* [22], Muthalagappan, *et al.* [28], Wilson, *et al.* [31], Christine and Kaldjian [36], Landmark, *et al.* [37], Lown, *et al.* [65], Saba, *et al.* [69], Charles, *et al.* [74], LeBlanc, Kenny, O'Connor, *et al.* [76], Peek, Quinn, Gorawara-Bhat, *et al.* [77]
	Teaching-learning	Elwyn, *et al.* [15], Friedberg, *et al.* [19], Frosch, *et al.* [21], Montori, *et al.* [27], Muthalagappan, *et al.* [28], Truglio-Londrigan [29], van Roosmalen, *et al.* [33], Charles, *et al.* [56], Shalowitz and Wolf [60], Ford, *et al.* [68], Charles, *et al.* [74], Peek, *et al.* [77]
	The decision point	Charles, *et al.* [6], Friesen-Storms, *et al.* [20], Sacchi, *et al.* [35], Lown, *et al.* [65], Saba, *et al.* [69], Charles, *et al.* [74]
Action for shared decision-making	Taking action on the decision	Frosch, *et al.* [21], Légaré and Witteman [25], Montori, *et al.* [27], Truglio-Londrigan [63], Ford, *et al.* [68]
	Returning to the provider to re-evaluate the decision	Durif-Bruckert, *et al.* [13], Friedberg, *et al.* [19], Frosch, *et al.* [21], Montori, *et al.* [27], Truglio-Londrigan [63], Ford, *et al.* [68], Zoffmann, *et al.* [73]

**Box 1:** A case study of shared decision-making.

JT is a 30-year-old female who will be visiting her primary care nurse practitioner (NP) for her annual physical exam. JT has been a patient at the Family Health Center for 10 years. During this time, she has established a relationship with her NP built on mutual trust and respect. There have been many times in the past 10 years when JT has called her NP with additional questions about her care and treatment. This accessibility allows for open communication and information exchange to provide opportunities for further discussion, education, and ultimately decision making. In fact, one of the primary reasons that JT is comfortable with her NP is the give and take trusting relationship that has been established. During visits, the NP always asks JT about her own opinions and ideas about care and treatment options so they can engage in the shared decision-making process. During this annual visit, JT plans to discuss whether or not she will have genetic testing for breast cancer. JT has a strong family history of breast cancer including her cousin, grandmother, and mother. JT is also aware that her mother is positive for the breast cancer gene (BRCA) and because of this she has been struggling with the uncertainty of being tested herself. During her yearly physical, the NP assesses what JT currently knows. The NP then spends time with JT providing education about genetic testing, what it entails, and what the results may indicate including the risks, benefits, and value this additional knowledge may or may not add to future decisions. The NP offers guidance on additional sources of information that JT may access on her own to enhance her knowledge as well as any community resources that may be available. Furthermore, the NP offers additional guidance by reminding JT that she is available for additional discussion. JT takes the information offered by her NP and searches out additional genetic counselling, education, and support groups in the community. The following week JT calls the Family Health Center and requests to speak with her NP. During the follow up phone call, in an attempt to find balance, JT and the NP continue the discussion about having genetic testing and what a positive outcome would mean. The NP provides additional education about the different courses of action but reminds JT that the first decision that must be made is whether or not JT wishes to be tested given her family history. Ultimately, JT takes action and makes the decision to be tested. She notes that she already lives a life of uncertainty about whether or not she will ever be diagnosed with breast cancer. If JT tests positive she would be able to make some definitive changes in her lifestyle that could prevent breast cancer and also make critical testing decisions that could lead to early diagnosis. JT knowns she can return to her NP at any time to assist in any uncertainty that may result from this decision. JT believes that the relationship with her NP and the inviting nature of the Family Health Center fosters a patient-centered culture necessary for shared decision-making.

## LIST OF ABBREVIATIONS

CENTRAL: Cochrane Central Register of Controlled Trials
EBP: Evidence-based practice
JBI-SUMARI: Joanna Briggs Institute System for the Unified Management, Assessment and Review of Information
SDM: Shared Decision-making
