# Supplementary material for: Shared Decision-Making for Nursing Practice: An Integrative Review
Source: Open Nurs J. 2018 Jan 22;12:1–14. doi: 10.2174/1874434601812010001 (PMC5806202; doi:10.2174/1874434601812010001)
Supplement: Supplementary file 1 [file TONURSJ-12-1_SP.pdf]

Send Orders for Reprints to reprints@benthamscience.ae

BENTHAM OPEN

CrossMark

The Open Nursing Journal

Supplementary Material

Content list available at: www.benthamopen.com/TONURSJ/

DOI: 10.2174/1874434601812010001

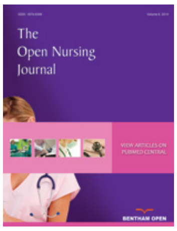

Shared Decision-Making for Nursing Practice: An Integrative Review

Marie Truglio-Londrigan[1,*] and Jason T. Slyer[2]

[1]*Pace University, College of Health Professions, Lienhard School of Nursing 861 Bedford Road Pleasantville, NY 10570, USA*

[2]*Clinical Assistant Professor, Pace University, College of Health Professions, Lienhard School of Nursing 163 William Street, 5th Floor New York, NY 10036, USA*

Received: October 13, 2017 Revised: December 16, 2017 Accepted: December 25, 2017

SUPPLEMENTARY MATERIALS

Supplemental Table (**1**): Critical appraisal results of included articles as well as a list of articles excluded based on methodological quality and the reason for exclusion.

Supp Table 1. Critical appraisal of included articles (n = 52).

Critical appraisal of included articles (n = 52).

**Experimental Studies (n = 5)**											
**Reference**	**Design**	**Groups randomly assigned**	**Participants blinded to treatment allocation**	**Allocation of treatment groups concealed from allocator**	**Outcomes of people who withdraw described and included in analysis**	**Outcome assessor blinded to treatment allocation**	**Control and treatment groups comparable at entry**	**Groups treated identically**	**Outcomes measured the same for all groups**	**Reliable outcome measures**	**Appropriate statistical analysis**
Bernhard et al. (2012)	RCT	U	U	U	N	U	Y	Y	Y	Y	Y
Bieber et al. (2006)	RCT	U	Y	U	Y	U	Y	Y	Y	Y	Y
Tinsel et al. (2013)	RCT	U	Y	N	Y	N	Y	Y	Y	Y	Y
van Roosmalen et al. (2004)	RCT	Y	N	N	Y	N	Y	Y	Y	Y	Y
Wilson et al. (2010)	RCT	Y	U	Y	N	U	Y	Y	Y	Y	Y
**Cohort Studies (n = 2)**											
Deinzer et al. (2009)	Cohort study	Y	U	Y	N	Y	Y	N	Y	Y	
Mandelblatt et al. (2006)	Cohort study	Y	Y	Y	N	Y	Y	N	Y	Y	
**Quantitative descriptive studies (n = 10)**											
Bot et al. (2014)	Non-experimental, correlational design	N	Y	Y	Y	NA	Y	Y	Y	Y	
Charles et al. (2004)	Cross sectional survey	N	Y	N	Y	NA	Y	NA	Y	Y	
Durif-Bruckert et al. (2015)*	Cross-sectional survey	N	Y	Y	Y	NA	Y	NA	Y	Y	

Suppl. Table 1 contd.....

**Experimental Studies (n = 5)**											
**Reference**	**Design**	**Groups randomly assigned**	**Participants blinded to treatment allocation**	**Allocation of treatment groups concealed from allocator**	**Outcomes of people who withdraw described and included in analysis**	**Outcome assessor blinded to treatment allocation**	**Control and treatment groups comparable at entry**	**Groups treated identically**	**Outcomes measured the same for all groups**	**Reliable outcome measures**	**Appropriate statistical analysis**
Glass et al. (2012)	Cross-sectional survey	Y	U	N	Y	NA	Y	NA	Y	Y	
Isaacs et al. (2013)	Cross-sectional survey	N	Y	N	Y	NA	Y	NA	Y	Y	
LeBlanc et al. (2009)	Exploratory descriptive	N	Y	N	Y	NA	Y	NA	Y	Y	
Légaré et al. (2011)	Cross-sectional survey	N	Y	N	Y	NA	Y	NA	Y	Y	
Ommen,et al. (2011)	Retrospective cross-sectional study	N	Y	Y	Y	NA	Y	NA	Y	Y	
Shabason et al. (2014)	Cross-sectional survey	N	Y	Y	Y	NA	Y	NA	Y	Y	
Smith et al. (2011)	Non-experimental, correlational design	U	Y	N	Y	NA	Y	NA	Y	Y	
**Qualitative Studies (n = 19)**											
		Meets Criteria (Yes [Y], No [N], Unclear [U], Not applicable [NA])									
Edwards et al. (2005)	Focus group	Y	Y	Y	Y	Y	N	N	Y	U	Y
Elwyn et al. (2001)	Qualitative descriptive	Y	Y	Y	Y	Y	N	N	Y	Y	Y
Ford et al. (2003)	Qualitative inquiry	Y	Y	Y	Y	Y	N	N	Y	U	Y
Friedberg et al. (2013)	Qualitative descriptive	Y	Y	Y	Y	Y	N	N	Y	Y	Y
Frosch et al. (2012)	Focus group	Y	Y	Y	Y	Y	N	N	Y	Y	Y
Lown et al. (2009)	Qualitative descriptive	Y	Y	Y	Y	Y	N	N	Y	Y	Y
Müller-Engelmann et al. (2011)	Qualitative exploratory	Y	Y	Y	Y	Y	N	N	Y	Y	Y
Peek et al. (2013)	Focus group	Y	Y	Y	Y	Y	Y	N	Y	Y	Y
Peek et al. (2010)	Phenomenological study	Y	Y	Y	Y	Y	Y	N	Y	Y	Y
Peek et al. (2009)	Phenomenological study	Y	Y	Y	Y	Y	Y	N	Y	Y	Y
Peek et al. (2008)	Focus group	Y	Y	Y	Y	Y	Y	N	Y	Y	Y
Saba et al. (2006)	Grounded theory	Y	Y	Y	Y	Y	N	N	Y	Y	Y
Shay and Lafata (2014)	Qualitative descriptive	Y	Y	Y	Y	Y	N	N	Y	Y	Y
Thorne et al. (2013)	Constant comparative analysis	Y	Y	Y	Y	Y	N	N	Y	Y	Y
Towle et al. (2006)	Qualitative descriptive	Y	Y	Y	Y	Y	N	N	Y	Y	Y
Truglio-Londrigan (2013)	Qualitative descriptive	Y	U	Y	Y	Y	Y	Y	Y	Y	Y
Truglio-Londrigan (2015)	Qualitative descriptive	Y	U	Y	Y	Y	Y	Y	Y	Y	Y
Upton et al. (2011)	Qualitative descriptive	Y	Y	Y	Y	Y	N	N	Y	N	Y
Zoffmann et al. (2008)	Grounded theory	Y	Y	Y	Y	Y	Y	Y	Y	Y	Y
**Conceptual papers (n = 16)**											
Charles et al. (1997)		Y	Y	Y	Y	Y	Y	Y			

Suppl. Table 1 contd.....

**Experimental Studies (n = 5)**										
**Reference**	**Design**	**Groups randomly assigned**	**Participants blinded to treatment allocation**	**Allocation of treatment groups concealed from allocator**	**Outcomes of people who withdraw described and included in analysis**	**Outcome assessor blinded to treatment allocation**	**Control and treatment groups comparable at entry**	**Groups treated identically**	**Outcomes measured the same for all groups**	**Reliable outcome measures**	**Appropriate statistical analysis**
Charles et al. (1999)		Y	Y	Y	Y	Y	Y	Y			
Charles et al. (2006)		Y	Y	Y	Y	Y	Y	Y			
Christine and Kaldjian (2013)		Y	Y	Y	Y	Y	Y	Y			
Friesen-Storms et al. (2015)		Y	Y	Y	Y	Y	Y	Y			
Hain and Sandy (2013)		Y	Y	Y	Y	Y	Y	Y			
Hess et al. (2015)		Y	Y	Y	Y	Y	Y	Y			
Lally et al. (2011)		Y	Y	Y	Y	Y	Y	Y			
Landmark et al. (2015)		Y	Y	Y	Y	Y	Y	Y			
Légaré and Witteman (2013)		Y	Y	Y	Y	Y	Y	Y			
Montori et al. (2006)		Y	Y	Y	Y	Y	Y	Y			
Muthalagappan et al. (2013)		Y	Y	Y	Y	Y	Y	Y			
Sacchi et al. (2015)		Y	Y	Y	Y	Y	Y	Y			
Shalowitz and Wolf (2004)		Y	Y	Y	Y	Y	Y	Y			
Siminoff and Step (2005)		Y	Y	Y	Y	Y	Y	Y			
White et al. (2003)		Y	Y	Y	Y	Y	Y	Y			

*Mixed Method study-also met qualitative criteria with 8 yes.
Note. Randomized controlled trial [RCT], Yes [Y], No [N], Unclear [U], Not applicable [NA]

Excluded Studies

Stevenson FA. (2003). General practitioners' views on shared decision making: A qualitative analysis. Patient Educ Couns. 2003; 50(3): 291-3.

Reason for exclusion: did not have an adequate representation of participant's voices.

Davis RE, Dolan G, Thomas S, Atwell C. Mead D, Nehammer S, *et al.* Exploring doctor and patient views about risk communication and shared decision-making in the consultation. Health Expect. 2003; 6(3), 198-207.

Reason for exclusion: did not have an adequate representation of participant's voices.

Edwards A, Elwyn G. Inside the black box of shared decision making: distinguishing between the process of involvement and who makes the decision. Health Expect. 2006; 9(4), 307-20. doi:10.1111/j.1369-7625.2006.00401.x

Reason for exclusion: incongruity between the research methods and the philosophical perspective, stated objectives, data collection methods, and interpretation of the results.

Supplemental Table (**2**): Description of included articles, including aims/objectives and summary of findings.

Suppl Table 2. Critical appraisal of included articles (n = 52).

Appendix 2. Description of included articles (n=52)			
**Quantitative Studies (n=16).**			
**Author/Country/ Method**	**Aims/Objectives**	**Participants**	**Findings**
Bernhard *et al.* (2012)<br>Australian/New Zealand<br>Switzerland/<br>German/Austria<br>-RCT	To identify the beneficial impact on decisional conflict in patients of physicians trained in SDM.	Swiss/German/ Austrian: physicians n=42; patients n=390.<br>Australian/New Zealand: physicians n=21; patients n=304.	-No overall effect on patient decisional conflict.
Bieber *et al.* (2006)<br>Germany<br>-RCT	To investigate the effects of a SDM intervention on physician-patient interactions and health outcomes.	Fibromyalgia patients from an outpatient university setting SDM group n=34; information group n= 33.	-Quality of physician-patient interaction noted to be higher in the SDM group.
Bot *et al.* (2014)<br>United States<br>-Non-experimental, correlational design	To assess predictors of patient satisfaction, ratings of the provider's informed SDM, and disability among patients with orthopedic pain complaints.	130 adult patients with non-traumatic painful upper extremity conditions were included.	-Patients identified that a moderate level of informed SDM was practiced by orthopedic surgeons.<br>-Health anxiety was found to be a significant predictor of both patient satisfaction and informed SDM.
Charles, Gafni, and Whelan (2004)<br>Canada<br>-Cross sectional survey	To explore the extent to which breast cancer specialists report practicing SDM with their patients, their comfort level with SDM, and perceived barriers and facilitators.	232 surgeons and 102 oncologists.	-Physicians identified comfort with the SDM approach.<br>-Barriers include lack of time, patient anxiety, patient lack of information and/or misinformation, and patient unwillingness or inability to participate.<br>-Facilitators include patient's emotional readiness, support, information, and trust in the physician.
Deinzer, Veelken, Kohnen, and Schmieder (2009)<br>Germany<br>-Cohort study	To assess whether patient empowerment in the management of hypertension improved with SDM.	SDM group n=40; control group n=40.	-SDM did not improve management.
Glass *et al.* (2012)<br>United States<br>-Cross-sectional survey	To examine the relationship between SDM and satisfaction with decisions.	488 patients were recruited from a health research volunteer registry.	-SDM is associated with satisfaction with decisions primarily noted in: understanding information, treatment preferences elicitation, and weighing options.
Isaacs *et al.* (2013)<br>United States<br>-Cross-sectional survey	To assess the relationship between older adults' perceptions of SDM in the selection of analgesic to take at home for acute musculoskeletal pain, patient satisfaction with analgesic, and changes in pain scores.	111 individuals age 65 and older who visited an emergency department.	-Patients who participated in the decisions were more likely to report satisfaction with the analgesic and a decrease in pain.
LeBlanc, Kenny, O'Connor, and Légaré (2009)<br>Canada<br>-Before and after study	To explore the effect of feeling uninformed, unclear values, inadequate support, and the perception that an ineffective decision has been made on one's own outcome and on the other person's outcome.	Secondary analysis of data from 112 dyads of physicians and patients.	-Patient and physician uncertainty o is influenced by personal deficits and by the deficits of the other member of the dyad.
Légaré *et al.* (2011)<br>Canada<br>-Cross-sectional survey	To assess the willingness of women and their family physicians to engage in SDM in regards to prenatal Down-syndrome screening.	109 pregnant women and 41 family physicians.	-A woman's attitude, significant others, self-efficacy, perceived moral correctness, and their family physician attitude influence willingness to engage in SDM.

Suppl. Table 2 contd.....

Appendix 2. Description of included articles (n=52)			
**Quantitative Studies (n=16).**			
**Author/Country/ Method**	**Aims/Objectives**	**Participants**	**Findings**
Mandelblatt, Kreling, Figeuriedo, and Feng (2006)<br>United States<br>-Prospective cohort study	To describe patient and physician determinants of SDM in older women with breast cancer and evaluate whether SDM is associated with treatment patterns or short-term outcomes of care.	718 women 67 years of age and older treated for early stage breast cancer in 29 different sites from five geographic regions.	-Younger women reported higher SDM than women 75 years of age and older.<br>-Women accompanied to consultations reported higher SDM than women who went to appointments alone.<br>-Women who reported having a treatment choice reported higher SDM than those who felt they did not have a choice.<br>-SDM was associated with satisfaction.
Ommen, Thuem, Pfaff, and Janssen (2011)<br>Germany<br>-Retrospective cross-sectional study	To investigate the relationship between social support (emotional and informational), SDM, and inpatients' trust in physicians.	2,197 patients who received inpatient treatment.	-A relationship between SDM behaviors, social support age, socioeconomic status, gender, and patient's trust in physicians were noted.
Shabason, Mao, Frankel, and Vapiwala (2014)<br>United States<br>-Cross-sectional survey	To assess the prevalence of SDM and the perception of control in treatment decisions among patients receiving radiation therapy, and to explore the relationship between a patient's desire for and perception of control during radiation therapy on satisfaction, anxiety, depression, and fatigue.	305 patients undergoing radiation therapy for a diagnosis of cancer.	-Approximately 1/3 of the patients experienced SDM, 1/3 perceived control in treatment decisions, 3/4 reported being satisfied and 1/3 reported feeling very satisfied with their plan of care.<br>-Patient satisfaction was associated with perceived SDM and patient-perceived control.<br>-Increase in anxiety, depression, and fatigue was reported from patients who desired but did not perceive control.
Smith *et al.* (2011)<br>Australia<br>-Non-experimental, correlational design	To assess the relative impact of cognitive and emotional aspects of SDM on patient outcomes.	20 clinicians with 55 consultations from cancer centers with patients diagnosed with early stage breast cancer.	-Emotional relating and SDM behaviors in a consultation are related to patient outcomes.<br>-High levels of emotional blocking behavior by the physician were related to decisional conflict.
Tinsel *et al.* (2013)<br>Germany<br>-Clustered RCT	To implement an evaluated SDM training program for general practitioners within the context of hypertension treatment.	1120 patients from 36 general practices.<br>Intervention group: 17 practices with 552 patients.<br>Control group: 19 practices with 568 patients.	-No significant effect from the SDM training on patient outcomes.
van Roosmalen *et al.* (2004)<br>Netherlands<br>-RCT	To evaluate a SDM intervention for BRACA1/2 mutation carriers who have to make a choice between screening and prophylactic surgery for breasts and/or ovaries.	88 women (intervention n=44; control n=44), either affected or unaffected with breast and/or ovarian cancer that decided to undergo DNA testing.	-The SDM group had less intrusive thoughts about cancer in the family, better general health, and was less depressed.<br>-The SDM group held stronger treatment preferences and more strongly agreed to having weighed the pros and cons.
Wilson *et al.* (2010)<br>United States<br>-RCT	To compare two decision-making models (SDM and clinical decision making) on asthma controller medication adherence and clinical outcomes in adults with poorly controlled asthma.	612 adults with poorly controlled asthma (SDM group n=204; clinical decision-making group n= 204; usual care control group n=204).	-SDM and negotiating treatment decisions significantly improves adherence to asthma pharmacotherapy and clinical outcomes.
**Mixed methods studies (n=1)**			
**Author/Country/Method**	**Aims/objective**	**Participants**	**Findings**

Suppl. Table 2 contd.....

**Appendix 2. Description of included articles (n=52)**			
**Quantitative Studies (n=16).**			
**Author/Country/ Method**	**Aims/Objectives**	**Participants**	**Findings**
Durif-Bruckert *et al.* (2015)<br>France<br>-Mixed Method	To understand patients' perceptions on SDM.	Quantitative analysis conducted on a sample of 132 early-stage breast cancer patients to determine their perceptions of information given to them and their participation in decisions.<br>Qualitative semi-structured interviews with 14 early-stage breast cancer patients in parallel with observed patient-physician consultations.	Quantitative analysis revealed:<br>-84% of patients stated they wanted to participate in decisions on their surgery.<br>-81% of patients considered that they did actually participate in decision-making.<br>-98% of patients said they were satisfied.<br>Qualitative analysis revealed:<br>-Barriers to participation included: emotional shock and uncertainty about treatment outcomes.<br>-Facilitators to participation included: trust in surgeon.
**Qualitative Studies (n=19)**			
**Author/Country/Method**	**Aims/objective**	**Participants**	**Findings**
Edwards *et al.* (2005)<br>United Kingdom<br>-Focus group	To identify the experiences and views of professionals skilled in SDM and risk communication.	20 general practitioners, who previously participated in a randomized controlled trial where the intervention was training in SDM.	-Training in SDM was positive in involving the patient, the relationship, and patient satisfaction.
Elwyn *et al.* (2001)<br>United Kingdom<br>-Qualitative descriptive	To examine the communication strategies of general practitioners attempting to involve patients in treatment or management decisions.	4 general practitioners who taped consultations with patients with the specific intent to involve patients in the process of SDM.	-Findings identify that communication strategies that facilitate the concept of equipoise are necessary.
Ford, Schofield, and Hope (2003)<br>United Kingdom<br>-Qualitative inquiry	To identify the elements and skills required for successful evidence-based patient choice.	11 general practitioners, 10 hospital physicians, 5 nurses, 11 academics, and 8 lay individuals.	Six themes emerged:<br>-Research evidence/medical information<br>-Physician patient relationship<br>-Patient's perspective<br>-Decision-making process<br>-Time issues<br>-Establishing nature of the problem.
Friedberg, Van Busum, Wexler, Bowen, and Schneider (2013)<br>United States<br>-Qualitative descriptive	To understand how delivery systems can implement SDM.	Semi-structured interviews of 23 individuals from 8 primary care sites.	Barriers noted included:<br>-Overworked providers<br>-Insufficient provider training on SDM<br>-Clinical information systems inability to prompt provider when decision aids may be useful and tracking patients through the SDM process.<br>Solutions offered include:<br>-Automatic triggers in health care systems via the use of information systems and engaging team members in the process of care.
Frosch, May, Rendle, Tietbohl, and Elwyn (2012)<br>United States<br>-Focus group	To explore why some patients are reluctant to engage in collaborative discussions about choices in health care with their physicians.	6 focus groups with 48 people from primary care practices.	Findings:<br>-Patients felt the need to conform to socially sanctioned roles of the "good" patient by not questioning or challenging their physician.<br>-Patients felt that an authoritarian physician did not respect them.<br>-Patients described how they did not have the opportunity to ask questions, voice concerns, or seek guidance.<br>-Patients identified the need to bring family or friends to clinical situation for social support.

Suppl. Table 2 contd.....

**Appendix 2. Description of included articles (n=52)**			
**Quantitative Studies (n=16).**			
**Author/Country/ Method**	**Aims/Objectives**	**Participants**	**Findings**
Lown, Clark, and Hanson (2009)<br>United States<br>-Qualitative descriptive	To explore how patients and physicians describe attitudes and behaviors that facilitate SDM.	85 patients and physicians in primary care settings.	Themes:<br>-Patient and physician act in a relational way.<br>-Patient understands and expresses feelings, preferences, and information about self and the physician explores patients' feelings, preferences, and information about self via exploration.<br>-Patient and physician discuss information and options.<br>-Patient and physician seek information, support, and advice.<br>-Patient and physician share control and negotiate decisions.<br>-Patient acts on behalf of self and the physician acts on behalf of the patient.
Müller-Engelmann, Keller, Donner-Banzhoff, and Krones (2011)<br>Germany<br>-Qualitative exploratory	To determine which treatment situations were suitable for SDM.	12 general practitioners, 15 patients, and 13 health administration and research professionals.	Factors that influence SDM:<br>-Minor or severe disease<br>-Acute or chronic disease<br>-Prevention or therapy<br>-Urgency of immediate medical action<br>-Single or multiple therapeutic options<br>-Adverse effects of invasiveness<br>-Evidence of efficacy<br>-Characteristics of the patient.
Peek *et al.* (2013)<br>United States<br>-Focus group	To explore patient trust in physicians and its relationship to SDM among African-Americans with diabetes.	24 in-depth interviews,<br>5 focus groups	Themes:<br>-Race and trust.<br>-Interpersonal relationship aspect of trust: physician racial bias and cultural discordance negatively affect relationships.<br>-Medical skills/technical competence aspects of trust.<br>-Influence of SDM on patient trust: physician SDM behaviors are facilitators of patient trust.<br>-Influence of patient trust on SDM: patient trust leads to more SDM preferences.

Suppl. Table 2 contd.....

**Appendix 2. Description of included articles (n=52)**			
**Quantitative Studies (n=16).**			
**Author/Country/ Method**	**Aims/Objectives**	**Participants**	**Findings**
Peek *et al.* (2010) United States -Phenomenological study	To explore patient perceptions of how race may influence SDM among African-American patients and their physicians.	24 in-depth interviews, 5 focus groups	Themes from in-depth interviews: -Relevance of race: race does not influence patient/provider communication or SDM. -Mechanisms for race influencing SDM: surrounding cultural discordance or cultural differences as problematic. -Influence of race on SDM: race-related issues may affect SDM behaviors of patients by being less forthcoming, speaking up or question authority of physician, and less likely to adhere to treatments. Themes from focus groups: -Relevance of race: race does influence patient/physician unpleasant communication. -Mechanisms for race influencing SDM: physician bias/discrimination and/or cultural discordance. -Influence of race on SDM: physicians less likely to provide information and less likely to listen and "talk down to" patients.
Peek *et al.* (2009) United States -Phenomenological study	To explore the barriers and facilitators of SDM among African-Americans with diabetes.	24 semi-structured interviews, 5 focus groups	Themes revealed: -Patient factors: patient/physician power imbalance, health literacy, trust, family experiences, fear and denial, and self-efficacy. -Physician factors: information-sharing and patient education, validation of health concerns, physician medical knowledge/technical skills, accessibility and availability, and interpersonal skills. -Patient/physical power imbalance.
Peek *et al.* (2008) United States -Focus group	To investigate how SDM is defined by African-American patients with diabetes and compares patients' conceptualization of SDM with Charles' model Peek *et al.* (2013).	24 semi-structured interviews, 5 focus groups	Conceptualization: -Shared decision-making: equal relationship and having a say. -Information sharing: understandable communication in non-technical language. -Physician recommendations. -Decision-making (passive patients; shared patients; autonomous patients).
Saba *et al.* (2006) United States -Grounded theory	To examine SDM and the subjective experience of partnerships for patients and physicians in primary care	10 physicians and 18 patients in 3 clinics.	Archetypes of engagement in decision making: -Full engagement -Simulated engagement -Assumed engagement -Non-engagement

Suppl. Table 2 contd.....

Appendix 2. Description of included articles (n=52)			
**Quantitative Studies (n=16).**			
**Author/Country/ Method**	**Aims/Objectives**	**Participants**	**Findings**
Shay and Lafata (2014)<br>United States<br>-Qualitative descriptive	To develop a conceptual model of patient defined SDM and understand what leads patients to label a specific decision-making process as shared.	23 patients in primary care settings	Patients defined SDM to include:<br>-Both physician and patient share information.<br>-Both are open-minded and respectful.<br>-Patient self-advocacy.<br>-Personalized physician recommendation.<br>-Long term trusting relationship.
Thorne, Oliffe, and Stajduhar (2013)<br>Canada<br>-Constant comparative analysis	To contribute to the evolving dialogue on optimizing cancer care communication through systematic analyzes of patients' perspectives.	60 cancer patients with diverse experiences were interviewed and audiotaped.	Communication barriers and facilitators of SDM:<br>-Focusing attention on the tone and setting of the environment.<br>-Attitudinal climate within the consultation.<br>-Specific approaches to handling information.<br>-Critical messaging around hope.
Towle, Godolphin, Grams, and Lamarre (2006)<br>Canada<br>-Qualitative descriptive	To investigate the practice, experiences, and views of family physicians as they attempt to implement informed and shared decision-making in practice.	6 family physicians received training on SDM and the information on the competencies for SDM.<br>198 data sets including: physician logs, patient satisfaction questionnaire, audiotapes, and group interview.	-Physicians viewed the training sessions as positive.<br>-Physicians noted a need for additional competences for SDM.
Truglio-Londrigan (2013)<br>United States<br>-Qualitative descriptive	To describe the experience of SDM in home-care from the nurse's perspective.	10 home-care nurses.	Themes uncovered:<br>-Begin where the patient is.<br>-Education for SDM.<br>-The village and SDM.<br>-Whose decision is it?
Truglio-Londrigan (2015)<br>United States<br>-Qualitative descriptive	To understand and describe the experience of SDM from the patient's perspective.	Six participants in home care settings	Themes uncovered:<br>-Creating the SDM experience.<br>-Carrying out the SDM experience.<br>-Carrying on the SDM experience.
Upton *et al.* (2011)<br>United Kingdom<br>-Qualitative descriptive	To investigate how nurses approach decision-making in relation to inhaler choice and long-term inhaler use.	20 nurses	Themes:<br>-Providing information and offering limited choice.<br>-Power and persuasion: nurses did consider patients as a partner; nurses identified that they held the power because of their clinical knowledge and often persuaded patients to agree with their recommendations.<br>-Sharing decisions to increase adherence: nurses view SDM as a tool to improve patient outcomes.<br>-Barriers of SDM: cost, time constraints.
Zoffmann, Harder, and Kirkevold (2008)<br>Denmark<br>-Grounded theory	To develop a theory on how patient-provider communication and reflection in the advanced field of diabetes care might lead to success SDM.	11 patients and 8 nurses from one inpatient unit and one day clinic at a university hospital.	A person-centered communication and reflection model was developed.

Conceptual Papers (n=16)		
**Author/Country/**	**Aims/objective**	**Findings**

Suppl. Table 2 contd.....

**Appendix 2. Description of included articles (n=52)**			
**Quantitative Studies (n=16).**			
**Author/Country/ Method**	**Aims/Objectives**	**Participants**	**Findings**
Charles, Gafni, and Whelan (1997)<br>Canada	To provide greater conceptual clarity about SDM and identify key characteristics of this model.		Characteristics of SDM noted:<br>-SDM involves at least two participants: the physician and patient.<br>-Both parties participate in the process of treatment decision-making.<br>-Information sharing is a prerequisite to SDM.<br>-A treatment decision is made and both parties agree to the decision.
Charles, Gafni, and Whelan (1999)<br>Canada	To revisit and add elements to an earlier conceptual framework on SDM Charles *et al.* (1997).		The revised framework:<br>-Identifies different analytic stages (information exchange, deliberation, deciding on treatment).<br>-Recognizes that the decision-making approach may change during the healthcare encounter.<br>-Identifies decision approaches that lie between the paternalistic, shared, and advocating models.<br>-Discusses practical applications.
Charles, Gafni, Whelan, and O'Brien (2006)<br>Canada	To discuss the influence of culture on the process of treatment decision-making and SDM in the physician-patient encounter.		-Cultural influences are important in SDM.<br>-Cultural expectations and values influence SDM.<br>-Decision aide tool development is important for assisting patients in SDM.<br>-Decision aide tools must be culturally sensitive.
Christine and Kaldjian (2013)<br>United States	To identify how much information about evidence physicians should communicate to patients to enable them to make informed decision.		The answer depends upon:<br>-Assessments of physicians, preferences of patients, and the knowledge available in clinical situations.<br>-Provision of relevant and understandable information to patients.<br>-Communicating treatment options, eliciting patient preferences, and recognizing the authority of the patient.<br>-Dialogue between the patient and physician to promote SDM is to promote ethical principles.<br>-Communicating evidence is a necessary pillar of SDM.<br>-SDM involves balance of ethical principles.
Friesen-Storms, Bours, van der Weijden, and Beurskens (2015)<br>Netherlands	To discuss the relevance of SDM in chronic care and to suggest how it can be integrated with evidence-based practice in nursing.		Chronic care warrants SDM and inviting the patient to participate in the decision-making process.<br>-SDM takes place within the context of evidence-based practice.<br>-Attributes to be aware of include: levels of research and corresponding evidence, intervention options available, burden of side effects with each intervention option, impact on the patient, patient's values and beliefs, and availability of resources.
Hain and Sandy (2013)<br>United States	To discuss a patient-provider partnership model of care that supports SDM.		-The patient-provider partnership facilitates and supports SDM.<br>-Collaboration and engagement are essential in experiences when power is shared and where there is trust and mutual respect.<br>-The patient-provider partnership leads towards autonomy and empowerment.<br>-Informed patients are more likely to be autonomous and engaged in their care, leading to better health outcomes.<br>-Decision aids may be valuable tools to assist in this process.
Hess, Grudzen, Thomson, Raja, and Carpenter (2015)<br>United States	To highlight SDM within the emergency department.		Ethical implications of applying a practice based in SDM within the emergency department setting.<br>-Factors influencing the degree to which providers in emergency medicine apply SDM: patient factors, provider factors, contextual factors, strengths of evidence.

Suppl. Table 2 contd.....

**Appendix 2. Description of included articles (n=52)**			
**Quantitative Studies (n=16).**			
**Author/Country/ Method**	**Aims/Objectives**	**Participants**	**Findings**
Lally, Macphail, Palmer, Blair, and Thomson (2011)<br>United Kingdom	To explain what SDM is, why health professionals should increase patients' involvement in decisions about care, and what may facilitate it.		Components of SDM:<br>-Clarifying the decision to be made.<br>-Clarifying options available.<br>-Communicating risks and benefits of the treatment option.<br>-Exploring what is important to the patient.<br>Interventions to support SDM:<br>-Decision support tools.<br>-Provide information on options.<br>-Discussion to clarify what is important to individual patients.<br>Barriers to SDM:<br>- Limited time in practice settings.<br>- Limited professional skills.
Landmark, Gulbrandsen, and Svennevig (2015)<br>Norway	To describe how sharing in decisions are negotiated through epistemic and deontic resources.		There is a complex interplay that takes place via communication as patients and providers engage in the negotiation process leading to decisions.
Légaré and Witteman (2013)<br>Canada	To describe three elements of SDM: recognizing that a decision is required, understanding the best available evidence, and incorporating the patient's values and preferences into the decision.		Essential elements of SDM:<br>-Provider and patient must recognize that a decision is needed.<br>-Both parties understand the best available evidence.<br>-The decision considers the patient's values and preferences.<br>Barriers:<br>-Time<br>-Patient characteristics.<br>Implementing SDM requires:<br>-Provider education in the SDM approach.<br>-Practice needs to be reorganized around the principles of patient engagement.<br>-Patient-mediated interventions that facilitate the patient's interactions with the provider and the healthcare system.
Montori, Gafni, and Charles (2006)<br>Canada, United States	To discuss SDM with patients with chronic conditions.		-Treatment decisions in the chronic care setting are likely to require an active patient role; patients have a longer window of opportunity to make decisions and to revisit and reverse these decisions.<br>-Barriers to SDM in this context: inadequate appointment durations (time) and long periods between visits (time).
Muthalagappan, Johansson, Kong, and Brown (2013)<br>United Kingdom	To explore the ethical basis and empirical evidence around SDM in dialysis decisions among frail older adults with end stage renal disease.		-SDM is a continuum that depends on a patient's situation, preferences, and degree of understanding. Ethical principles dictate supporting a patient's decision.
Sacchi *et al.* (2015)<br>Italy	To promote the shift from a traditional, physician-centered, clinical decision process to a more personalized, patient-oriented SDM environment.		Personalized decision models can be used as a means to facilitate SDM by taking into account individual patient preferences.
Shalowitz and Wolf (2004)<br>United States	To discuss the conceptual framework of SDM and how obstacles to SDM for lower literacy patients may contribute to health-related harms.		SDM noted as three stages:<br>-Information flows are bi-directional.<br>-Deliberation concerning which course of action best "fits" the patient's life, ideas, values, and beliefs.<br>-Decisions that signify both parties agree on the treatment option.<br>-Limited literacy is a barrier.
Siminoff and Step (2005)<br>United States	To propose a model that identifies the communication process as a vehicle for decision making that is embedded within the physician-patient relationship and acknowledges it as a social process.		The communication model of SDM:<br>-Factor 1: Patient-physician communication antecedents.<br>-Factor 2: Communication climate.<br>-Factor 3: Treatment decisions.

Suppl. Table 2 contd.....

Appendix 2. Description of included articles (n=52)			
**Quantitative Studies (n=16).**			
**Author/Country/ Method**	**Aims/Objectives**	**Participants**	**Findings**
White, Keller, and Horrigan (2003) Unites States	To describe informed consent as a SDM process to assist patients in choosing a course of action.		Communication skills involved in facilitating SDM:<br>-Inquire—assess understanding and desired level of involvement.<br>-Inform—provide information.<br>-Inquire—assess understanding, reactions to information, and choice.

Note: RCT, Randomized controlled trial; SDM, Shared decision-making

© 2018 Truglio-Londrigan and Slyer.

This is an open access article distributed under the terms of the Creative Commons Attribution 4.0 International Public License (CC-BY 4.0), a copy of which is available at: https://creativecommons.org/licenses/by/4.0/legalcode. This license permits unrestricted use, distribution, and reproduction in any medium, provided the original author and source are credited.
